# Megapixel multi-elemental imaging by Laser-Induced Breakdown Spectroscopy, a technology with considerable potential for paleoclimate studies

**DOI:** 10.1038/s41598-017-05437-3

**Published:** 2017-07-11

**Authors:** J. O. Cáceres, F. Pelascini, V. Motto-Ros, S. Moncayo, F. Trichard, G. Panczer, A. Marín-Roldán, J. A. Cruz, I. Coronado, J. Martín-Chivelet

**Affiliations:** 1 Department Química Analítica, Fac. Ciencias Químicas, Universidad Complutense de Madrid, 28040 Madrid, Spain; 2 CRITT Matériaux Alsace, 19, rue de St Junien, 67305 Schiltigheim, France; 3 Institut Lumière Matière, UMR5306 Universitè Lyon 1-CNRS, Universitè de Lyon, 69622 Villeurbanne cedex, France; 4 Department Estratigrafía, Fac. Ciencias Geológicas, Universidad Complutense de Madrid, 28040 Madrid, Spain; 5 Institute of Paleobiology, Twarda 51/55, 00-818 Warsaw, Poland; 6 Instituto de Geociencias (CSIC, UCM), C/José Antonio Nováis 12, 28040 Madrid, Spain

## Abstract

Paleoclimate studies play a crucial role in understanding past and future climates and their environmental impacts. Current methodologies for performing highly sensitive elemental analysis at micrometre spatial resolutions are restricted to the use of complex and/or not easily applied techniques, such as synchrotron radiation X-ray fluorescence micro-analysis (μ-SRXRF), nano secondary ion mass spectrometry (nano-SIMS) or laser ablation inductively coupled plasma mass spectrometry (LA-ICP-MS). Moreover, the analysis of large samples (>few cm²) with any of these methods remains very challenging due to their relatively low acquisition speed (~1–10 Hz), and because they must be operated in vacuum or controlled atmosphere. In this work, we proposed an imaging methodology based on laser-induced breakdown spectroscopy, to perform fast multi-elemental scanning of large geological samples with high performance in terms of sensitivity (ppm-level), lateral resolution (up to 10 μm) and operating speed (100 Hz). This method was successfully applied to obtain the first megapixel images of large geological samples and yielded new information, not accessible using other techniques. These results open a new perspective into the use of laser spectroscopy in a variety of geochemical applications.

## Introduction

Reconstruction of past climate variability over a range of temporal or spatial scales has become a key task in the understanding of present-day and future climate. Since instrumental data are limited to the last few decades or centuries, reconstructions must be based on climate proxies gathered from natural archives of paleoclimate variability such as sediments, organisms skeletons, speleothems, tree rings, and ancient ice that can also be dated with resolutions that approach annual to sub-annual scales using a combination of radiometric and microstratigraphic methods^1, 2^. The paleoclimate record is enormous and still only partly explored, despite the notable advances of the last three decades. Rapid analysis of paleoclimatic proxies would enable providing quick response to the scientific and societal need for the characterization of past climate patterns of change, forcing factors, and their impacts. This task requires searching for new analytical techniques with the possibility to analyze relative large (dm scale) sample surfaces (i.e., to obtain longer and more continuous records of paleoclimate) with sub-millimetre-scale to sub-micrometre-scale resolution (i.e., to resolve in favourable paleoclimate archives inter-annual or seasonal changes^3–6^ that combine reasonable cost with high-speed analysis and simple sample preparation).

In this study, an all optical imaging technology, which fulfils these prerequisites, is proposed as a practical and useful tool for obtaining climate proxy records that is based on the elemental composition of geological samples. It is based on laser-induced breakdown spectroscopy (LIBS)^7^ and yields high-quality elemental imaging data with characteristics such as micrometric resolution, a ppm-scale limit of detection, and high scanning speed (100 pixels/s in the present paper). Although spatially-resolved LIBS analysis has already been demonstrated in the literature for heterogeneous materials, such as those used in paleoclimate research^8, 9^, we report for the first time to our knowledge the possibility of producing megapixel-size images of large samples. Other studies have demonstrated the capability and potential of LIBS imaging in various fields of application, such as biology^10^, geology^8^ and industry^11, 12^. The latter references demonstrated an instrumentation dedicated to the analysis of steel and oxidic inclusions with the possibility to scan several tens of square centimetre. In all the other cases, the probed surfaces typically had areas of 1 cm² or less and were measured with a lateral resolution ranging from 10 to 50 µm (i.e., the number of pixels shown in each image was 100 000 or less). Due to the requirements of paleoclimate data series reconstructions (i.e., analyses should cover large areas or transects with the highest possible spatial resolution), the application of this technology, and more generally all imaging methodologies, to such samples remains very challenging. Large sample sizes impose the need for a high scanning rate (in order to scan several cm² with micrometric resolution in a reasonable analysis time) and a reliable and fast auto-focusing system, as well as the capability to handle and analyse high amounts of data.

In this paper, we report the possibility of performing megapixel elemental imaging of relatively large samples. The all-optical tabletop instrumentation allows the study of several kinds of samples, including speleothems and biomineral skeletons such as those produced by corals, among others. This technique also offers attractive features such as the capacity for multi-elemental detection, and easy sample preparation, as well as a lack of restrictions on the shape or size of the samples and without the technical limitations imposed by vacuum operating conditions. This technology can be applied to essentially all types of geological and biological materials. Its application is herein demonstrated with two materials commonly used in paleoclimate time series reconstruction, specifically speleothems (calcium carbonate cave deposits) and corals (calcium carbonate skeletons). These results offer new insights into the use of LIBS imaging in Earth science applications and pave the way for numerous applications in the field of paleoclimatology.

## Preamble

Both speleothems and coral skeletons are commonly used for the reconstruction of paleoclimate series because they can be accurately age-dated by combining radiometric and microstratigraphic techniques, and because they can yield multi-proxy records. Most climate proxies that can be retrieved from these materials are geochemical, as changes in climate or environmental conditions occurring during their growth periods usually cause tiny but significant modifications in their elemental or isotopic compositions.

Stalagmites precipitate from seepage waters in caves, and usually consist of calcium carbonate, calcite being the most common mineral phase. Internally, stalagmites show internal micro-stratigraphy of diverse complexity^13^, which mainly result from the vertical accretion of growth layers through time, but also from the diagenetic overprint which takes place mostly during periods without speleothem growth. The mineralogical and petrological characterization of the internal micro-stratigraphy is a key task in any paleoclimate approach, and must precede and accompany any geochemical analysis^14^.

The distribution of trace elements through stalagmite stratigraphic records can, in the cases more favourable for paleoclimate reconstruction, reflect the changes that occurred in the chemistry of the parent waters and/or the physicochemical conditions of the original carbonate precipitation, and these can in turn depend on paleoclimate or paleoenvironmental conditions. When it is the case, changes in trace elements through the stalagmite as those yielded by the LIBS megapixel multi-elemental imaging, could be interpreted in terms of past climatic change^15^. The transfer functions between the proxy data and the climate variables are not however straightforward, and requires on noticeable effort in cave monitoring and physicochemical modelling. The distribution of trace-elements through stalagmites has a great potential in paleoclimate reconstruction^16^ that is however frequently masked by difficulty of separating the different controls influencing their different incorporation through time. In this sense, the trace-element images presented in this paper must be analysed in the petrologic and microstratigraphic context of the stalagmite, to discern the influence of crystallization pathways in the incorporation of trace elements to the stalagmite^17^.

The annual growth rate of such natural archives is rather variable in time and space and depends on the physicochemical conditions of mineral precipitation (e.g., drip rates, calcite supersaturation, and temperature), which usually occurs at rates of 0.01 to 2 mm per/year^18^. Changes in the composition of trace elements, such as Mg, Sr, and Ba, throughout the stalagmite are commonly interpreted as recording changes in paleoclimate variables such as temperature or rainfall^15, 19–22^. The paleoclimatic significance of these elements, however, is strongly dependent on each cave and the climatic context, and proxy calibration is a complex task that usually requires cave monitoring programmes^16, 22, 23^.

Scleractinian corals are capable of generating large structures of aragonite (calcium carbonate) produced by biological precipitation over periods of up to hundreds of years. Changes in some elemental ratios, such as Sr/Ca, Mg/Ca, Li/Mg, Li/Ca, U/Ca and Ba/Ca, through time are strongly dependent on seawater temperatures^6, 24–33^ and thus are commonly used as a proxy of sea surface temperatures (SST). In addition, other metal/Ca proxies, such as Mn/Ca, Cd/Ca, Y/Ca, and Pb/Ca, respond systematically to natural and anthropogenic changes in the environment^31, 34^. Corals are among the richest marine paleoclimate and paleoenvironmental archives and the best archetype for sclerochronology studies; many grow rapidly (>1 cm/year) and are long-lived (up to many centuries). Corals from tropical and temperate regions and from shallow to deep water environments have shown high-resolution (weekly–monthly) environmental records of SST, salinity (SSS) or variations in ocean chemistry such as the acidification of seawater or massive nutrient inputs^25, 26, 29, 32^.

## Results

### LIBS megapixel scanning

**Figure 1:**
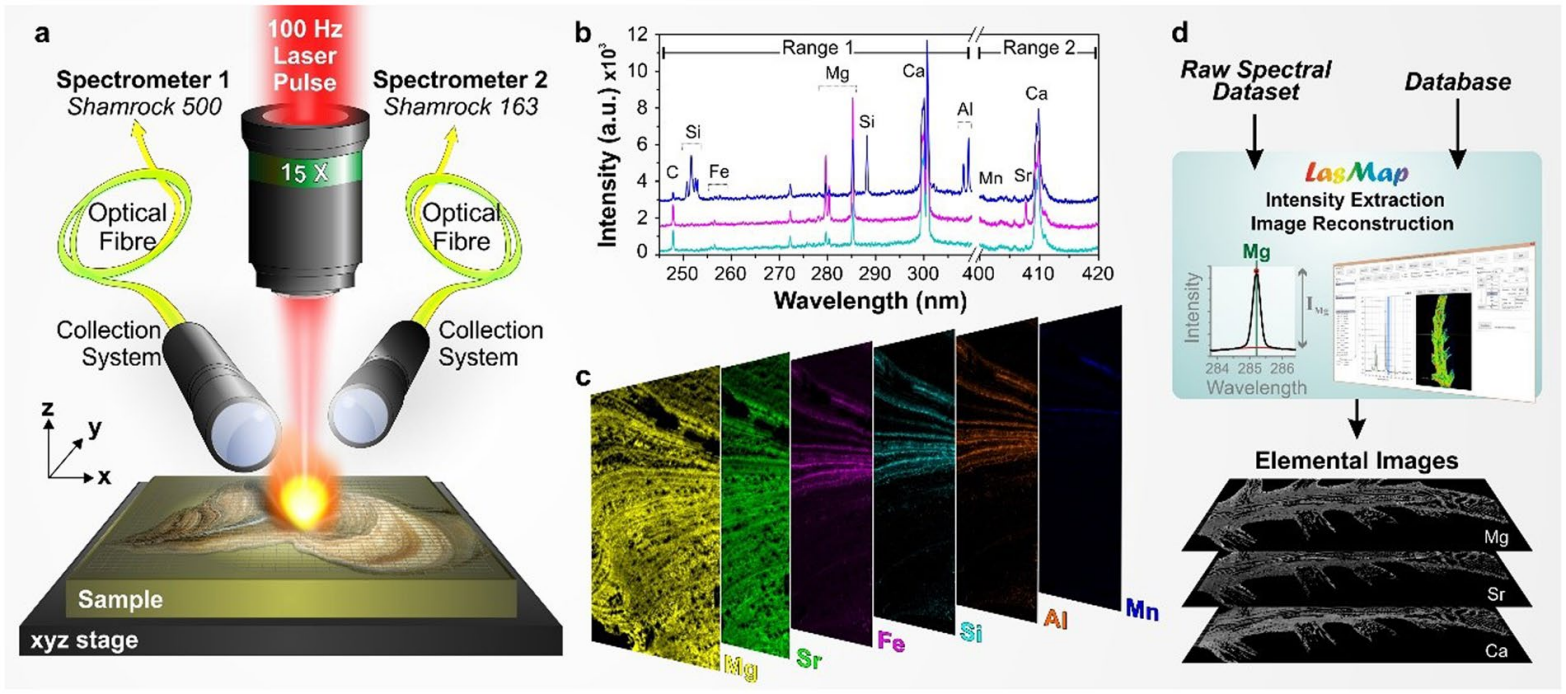
General protocol for LIBS imaging. (**a**) Schematic view of the LIBS instrument showing the major components: the microscope objective used to focus the laser pulse, the motorized platform supporting the sample and the two optical detection systems connected to different spectrometers via optical fibre bundles. (**b**) Sample single-pulse emission spectra covering spectral ranges between 245 and 310 nm (spectrometer 1) and between 400 and 420 nm (spectrometer 2) recorded in three different regions of the speleothem sample. These 3 spectra have been vertically shifted for better clarity. The characteristic emission lines of calcium (Ca), carbon (C), silicon (Si), iron (Fe), magnesium (Mg), aluminium (Al), manganese (Mn), and strontium (Sr) are clearly detected. (**c**) Sample relative-abundance images of Mg (red), Sr (cyan), Fe (violet), Si (yellow), Al (orange) and Mn (blue) represented using false colour scales for a 3000 × 1000 pixel sequence (resolution of 15 µm). (**d**) Principle of data analysis using the LasMap software.

The application of laser-induced breakdown spectroscopy (LIBS) for generating megapixel images from speleothems and corals should be focused on determining the distribution of multiple elements directly from large longitudinal sections with high spatial resolution. The experimental configuration mounted for these analyses has been given in the methods section and shown in Fig. 1a. This set-up and experimental conditions allowed achieving a minimum accessible resolution of approximately 10 µm, a value estimated from the size of the ablation craters that developed on calcium carbonate. The use of two spectrometers probing different spectral ranges allowed simultaneous detection of all the elements of interest (Fig. 1b). In the present study, we focused our attention on three main elements, magnesium (Mg), strontium (Sr) and calcium (Ca), although other elements, including sodium (Na), carbon (C), silicon (Si), iron (Fe), aluminium (Al), and manganese (Mn), were also detected. In our imaging configuration, laser-induced plasma was generated continuously while scanning the sample surface over the region of interest. The surface scanning was performed line by line in a raster scan mode with the use of a computer-controlled x-y-z stage. An emission spectrum was recorded for each sampling position. The travel range of the X motorized stage was 100 mm, while the travel range of the Y and Z stages was 50 mm. The X and Y ranges impose a limit on the maximum surface area that could be analysed in one scan (i.e., 50 cm²). The step size could be adjusted by changing the speed of the X stage over a range from a few µm to 100 µm (i.e., considering the maximum stage speed of 10 mm/s and the laser frequency rate of 100 Hz). We developed software in the LabVIEW environment that controlled all system components and allowed us to set automated sequences in order to scan regions of interest with the specified resolution.

After completion of the 2-D scanning, elemental images were created for each element of interest by selecting their specific emission lines (see Supplementary Table 1). Their relative abundance can be obtained and displayed in false-colour images, in which an arbitrary colour is assigned to each element (Fig. 1c). In the present manuscript, a typical imaging result represents approximately 3 million pixels (i.e., individual laser pulses) and an amount of data of approximately 50 GB (~25 GB/spectrometer). At a scanning rate of 100 Hz, the duration of such an analysis is approximately 8 hours (360 000 pixels/hour). Dedicated software was developed to handle the large amount of data collected and to extract elemental images from the raw spectra datasets (Fig. 1d). This software, named LasMap, includes different atomic and molecular databases that enable the fast identification of the emission lines observed in the raw spectra. After the line identification step, different algorithms can be used to extract the net line intensities from raw spectra. Once the spectral lines associated with the different elements of interest are selected, the software extracts the corresponding intensities spectrum by spectrum to create the elemental images associated with each element. This operation requires typically less than 7 minutes for a spectra dataset of 3 megapixels. To match the standard methodologies generally used in paleoclimate studies, the signal retrieved from the elements of interest (i.e., Mg and Sr) were normalized, pixel by pixel, by the Ca signal (i.e., the signature of the calcium carbonate phase). Although quantitative analysis was not required in the present case, the limits of detection of Mg and Sr were estimated at, respectively, 5 ppm and 7 ppm for single-pulse analyses using a reference sample of calcium carbonate performed in the laboratory.

The samples analysed herein include a ~25 cm long calcite speleothem collected from a cave (Cueva Mayor, Sierra de Atapuerca karst system, Spain) that is being investigated for paleoclimate studies and an ~8 cm-long sample from a recent cold-water scleractinian coral, *Dendrophyllia ramea*, that was retrieved from the Bay of Cadiz (on the Atlantic coast in southwestern Spain).

### Autofocus system

**Figure 2:**
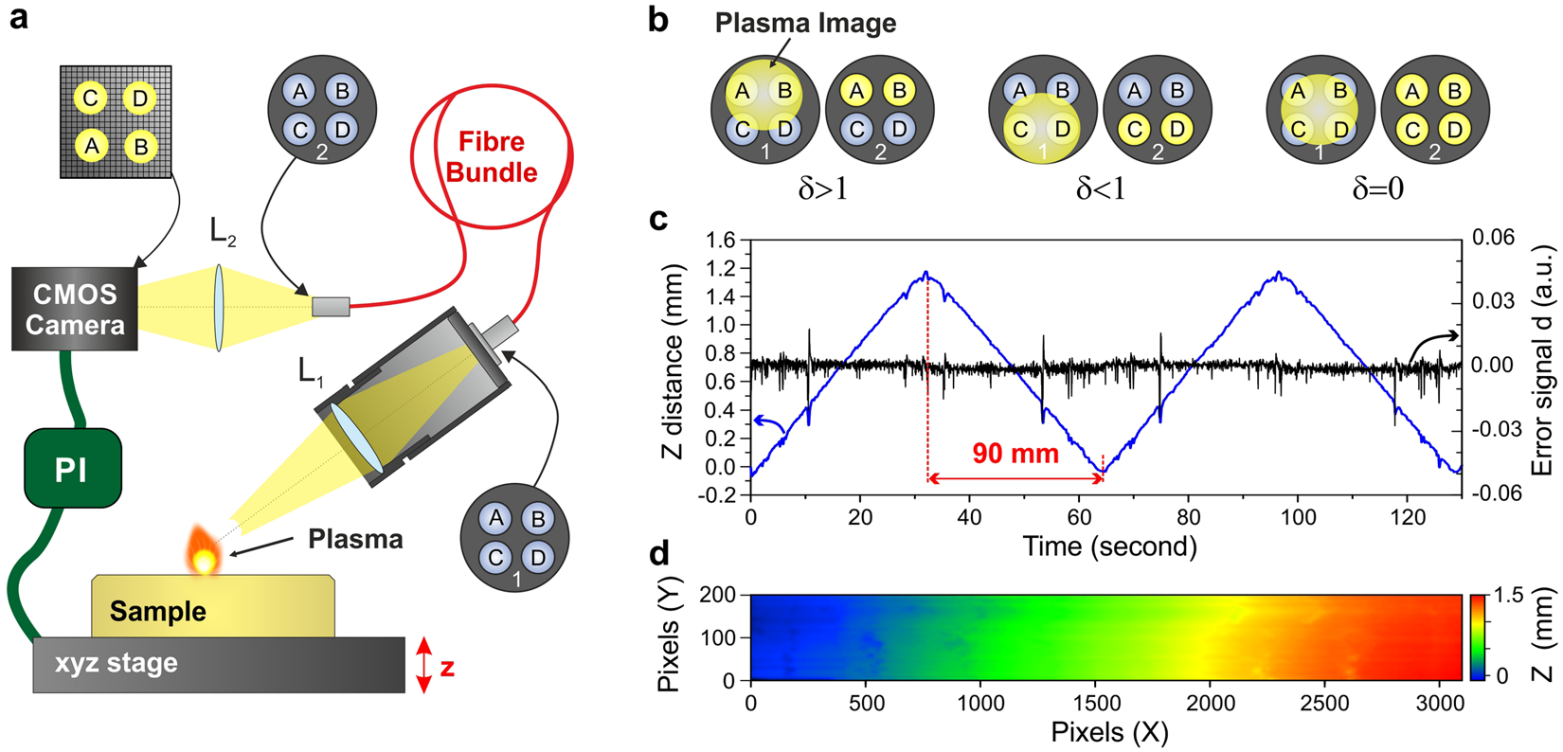
Autofocus system. (**a**) Schematic view of the autofocus system (PI: Proportional Integral locking servo). (**b**) Principle of the autofocus system and its influence on the error signal δ. (**c**) Vertical position of the sample (blue) and the associated error signal (black) for a 90-mm scan of the sample measured forward and backward twice. (**d**) Sample residual image showing the surface morphology and planarity of the sample during a 3100 × 200 scan (15 µm resolution). Note that in this example, a tilt of about 1.5 mm was deliberately set in the sample positioning to show the large accessible range of the autofocus correction.

The most critical aspect for imaging such large geological samples relies on accurate control of laser focusing. To guarantee reproducible ablations during the entire sequence scan, it is imperative to ensure a constant distance between the focusing objective and the sample surface. For such large samples (with surface areas greater than ~20 cm²), it is difficult, to obtain a sufficiently flat surface, even after rigorous preparation and polishing of the sample. In this work, we proposed a new type of autofocus applicable in any LIBS experiment. It relies on the basic idea of measuring and controlling the vertical position of the plasma emission at a high rate (Fig. 2a)^35^. For this purpose, the plasma is imaged on the entrance of a specific fibre bundle (connector 1 in Fig. 2a). The optical system used is similar to the ones used for light collection; the only difference is its angle (~55 deg.) with respect to the vertical axis. The fibre bundle consists of four fibres with a core diameter of 200 µm that are distributed symmetrically with respect to the centre of the connector (connectors 1 and 2 in Fig. 2a). The output of the fibre bundle is then imaged on a fast CMOS USB3 camera. This camera is triggered by the laser diode output signal and can typically provide 120 frame/second for a region of interest of 640 × 480 pixels. A fast algorithm, developed in the LabVIEW environment, extracts the global intensity collected by each of the four fibres in real time and calculates the following error signal:

1 $${\delta }_{z}=\frac{({I}_{A}+{I}_{B})-({I}_{C}+{I}_{D})}{({I}_{A}+{I}_{B})+({I}_{C}+{I}_{D})},\,$$

where *I*_*A*_ and *I*_*B*_ represent the total intensity collected in the two top fibres, and *I*_*C*_ and *I*_*D*_ represent that captured in the two bottom fibres (Fig. 2a). This error signal is positive if the sample surface (and thus the plasma) is high with regard to the reference position and negative in other case (Fig. 2b). This signal is then used in a proportional and integral (PI) system that provides the set point to the z-motorized stage. The decisive advantage of this autofocus system is based on its high operation speed. In the proposed configuration, this system operates at 100 Hz and can follow each laser pulse, although the correction is applied to the subsequent laser pulse in this autofocus concept. In addition, compared to conventional autofocus systems that use optical imaging (involving pointers or contrast imaging autofocus systems), this system has a much higher speed and better reliability because it is not dependent on sample properties (colour, transparency, etc). Finally, it should be mentioned that the autofocus speed could still be greatly improved by using a configuration including a quadrant photodiode and analogue PI locking, as well as a piezo system.

### Elemental imaging of a speleothem sample

**Figure 3:**
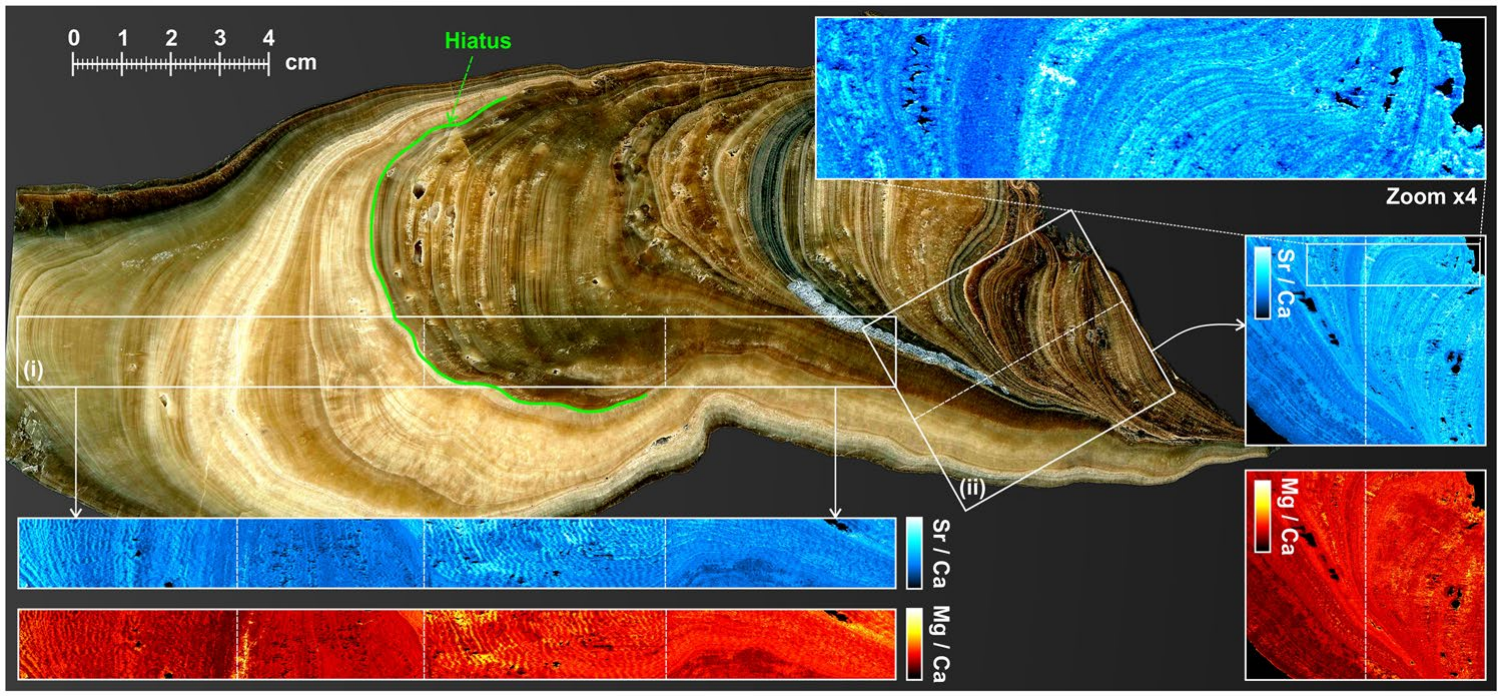
Speleothem imaging. Measurement (i): 15-µm resolution images of Sr (blue) and Mg (red) assembled from 4 successive sequences (shown by dot lines). The total scanned length corresponds to more than 16 cm, and the probed surface to ~24 cm². This analysis represents approximately 12 000 000 individual measurements (i.e., pixels). Measurement (ii): 15-µm resolution images of Sr and Mg for a ~5 × 4 cm sample surface. This analysis represents more than 9 000 000 pixels. A factor-of-four zoom is also shown for Sr. All the Sr and Mg images have been normalized by Ca. The green line indicates a major hiatal surface representing a time gap in calcite growth during speleothem development.

Megapixel LIBS imaging was first applied to a longitudinal section of stalagmite SLX-2B. This speleothem was chosen because of its provenance and properties. The sample was collected from a strongly isolated gallery where the temperature remains almost constant throughout the year (10.6 ± 0.1 °C), the relative humidity is close to 100%, and has no significant ventilation. The stalagmite, which is approximately 25 cm long, has been petrographically and microstratigraphically described in a previous work^36^. It consists of translucent calcite and is mostly formed by columnar fabrics, the columnar elongated and the columnar microcrystalline types being dominant. Its microstratigraphy is defined by sub-millimetre scale annual growth bands, and by a prominent non-depositional hiatus in its middle part (Fig. 3).

Two different LIBS images have been obtained from this sample. The first analysis was performed on the longitudinal axis of the speleothem (Fig. 3i) with a step size of 15 µm. This analysis covered a distance of approximately 16 cm × 1.5 cm, which represents a surface of ~24 cm² and a total number of pixels of more than 12 million. Because of the limited travel range of our motor (i.e., 10 cm), this analysis was conducted using 4 consecutive scans of ~3 megapixels each. These different scans were spliced together afterwards. The second imaging experiment was performed on the core of the speleothem (Fig. 3ii) and represented a probed surface of approximately 20 cm² (~5 cm × 4 cm) with approximately 9 million individual laser pulses. Two consecutive scans were required in this case because of technical limitations imposed by the performance of the computers used; these computers can record only 5 million spectra in a single scan. For both analyses, the Mg/Ca and Sr/Ca images are shown in Fig. 3. Magnification of sample region ii by a factor of four clearly shows the annual growth layers.

These images illustrate the resolution capabilities of the LIBS imaging method and show the spatial distribution of trace elements over large surfaces at micrometre scales. In the case of this study, compositional changes in Mg and Sr follow the patterns of the speleothem stratigraphy, a key aspect for demonstrating that compositional variations are not random and probably determined by temporal changes in hydrogeochemical conditions during stalagmite growth, i.e., variations in paleoenvironmental proxies^37^. It should be noted however, that any interpretation in this sense must be accomplished from an integrative point of view. A fundamental task is thus the integration of petrographic (optical) observations, and chemical results. The incorporation of trace elements in speleothem fabrics is dictated not only by the concentration of each element in the parent solution and the corresponding partition coefficients, but by a speleothem enrichment factor^38^, which accounts for extra-lattice incorporation of elements such as Sr.

This integrative work could allow the identification of compositional anomalies related to postdepositional geochemical overprints caused by diagenetic processes such as recrystallization, whose identification and elimination could be essential when looking for the original pristine chemical composition of the stalagmite. Non-altered areas of the speleothem are ideal for paleoclimate proxy extraction and U-series age dating.

**Figure 4:**
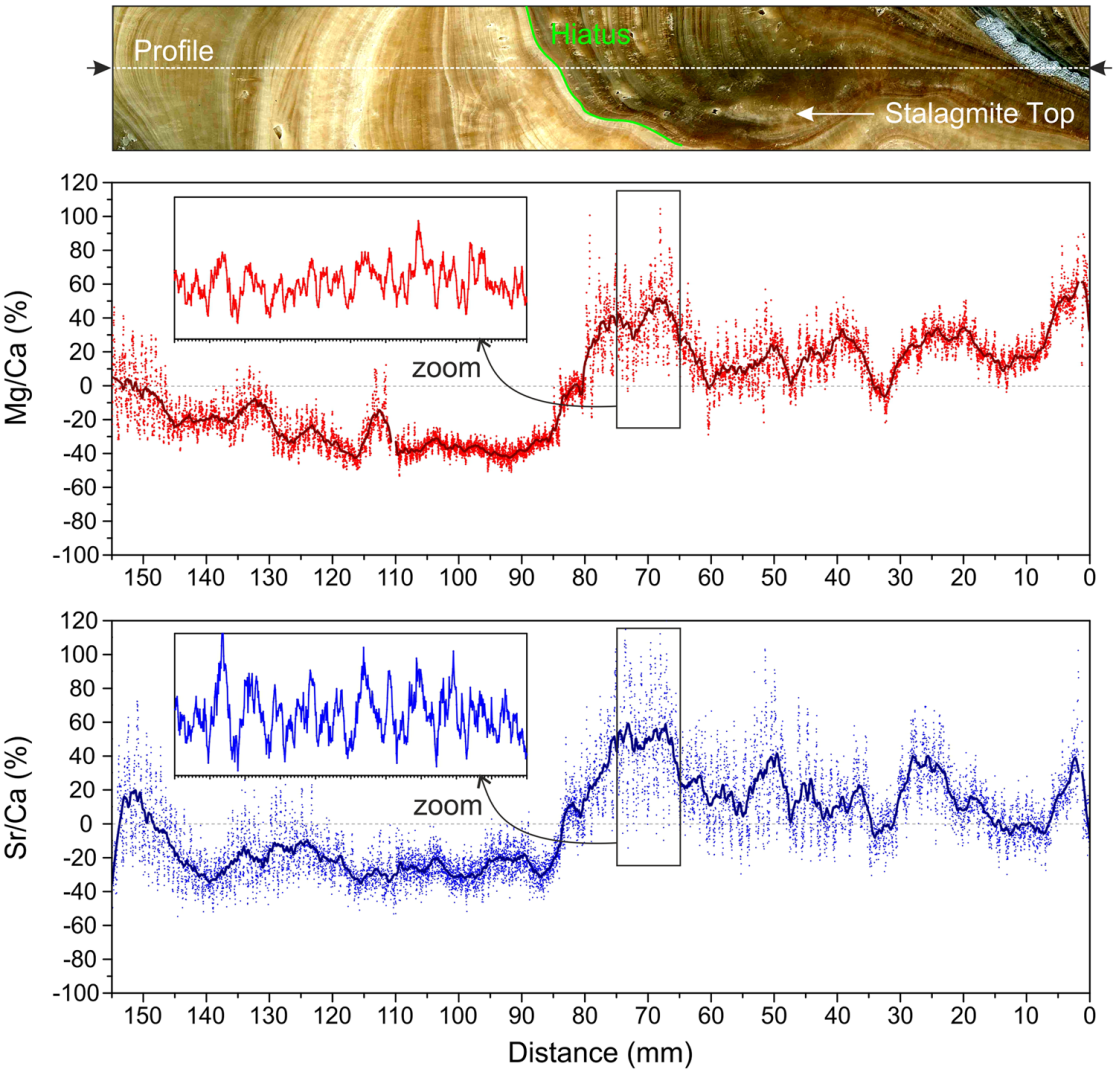
1-D data series showing relative variations in Sr/Ca and Mg/Ca (expressed in %) along the growing axis of the speleothem through the selected axial profile (indicated by the dotted line). Note that a non-depositional hiatus produces a major drop in the 1-D series of both the Mg/Ca and Sr/Ca ratios.

However, paleoclimate studies commonly focus on the construction of high-resolution time series, i.e., variations in one proxy through time. These 1-D data series can be easily extracted from LIBS images, which allow choosing the most favourable transects, usually those that follow the growth axis. Figure 4 shows the Mg/Ca and Sr/Ca relative changes (i.e., expressed in %) along the selected axial profile, including high-frequency fluctuations (corresponding to seasonal and inter-annual changes) as well as longer-term patterns with possible climatic significance. Note the presence of a major disruption in the 1-D series of both Mg/Ca and Sr/Ca ratios, which coincide with the non-depositional hiatus. That stratigraphic surface separates an area of greater Mg and Sr content and greater variability (below the hiatus) from another area characterized by lower values of both elements and a much lower dispersion in both the short- and the medium-term changes.

### Imaging of a coral sample

**Figure 5:**
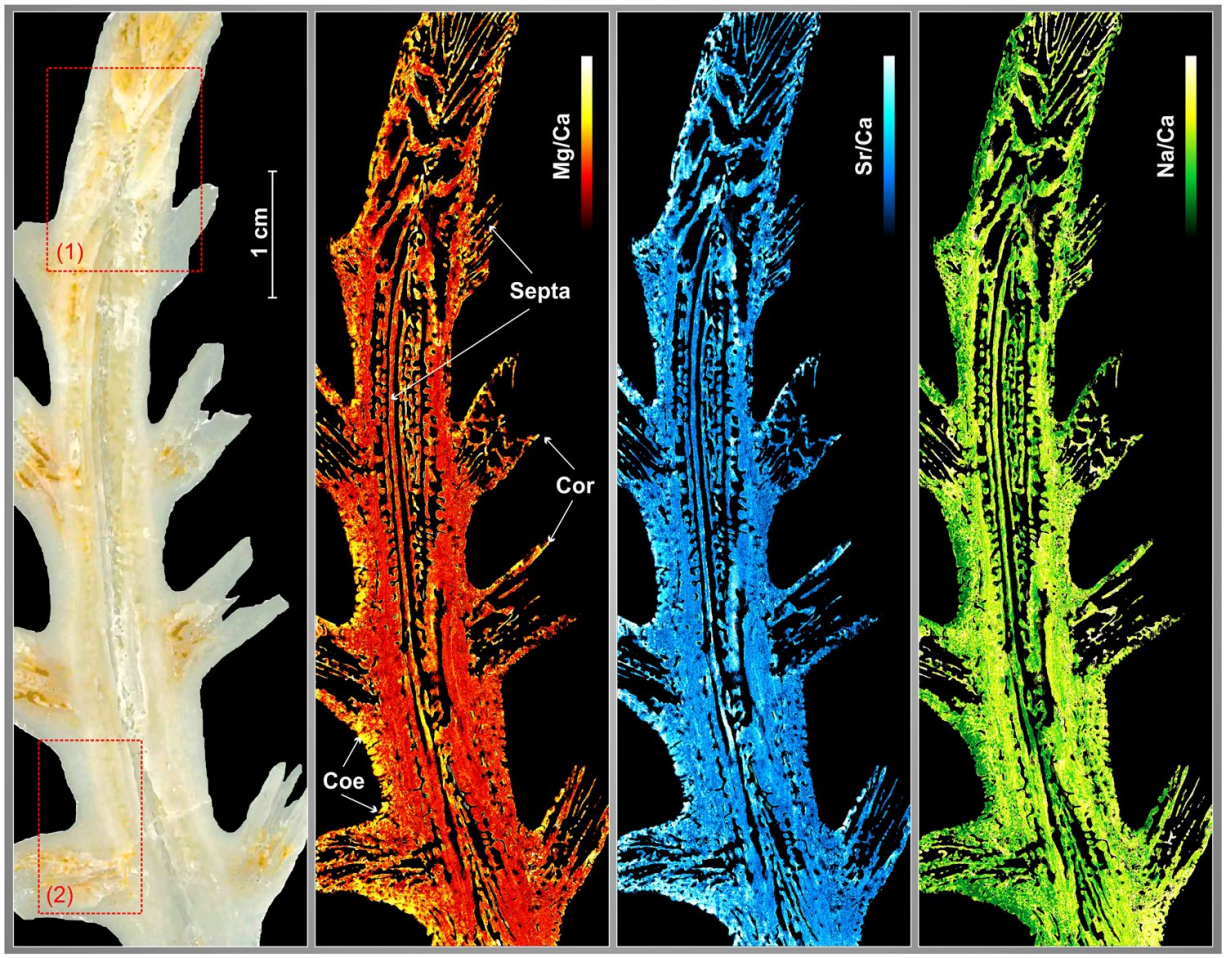
Optical 3300 × 1000 pixels image of *Dendrophyllia ramea* and its Mg/Ca, Sr/Ca and Na/Ca distributions with 25 µm resolution. The septa, coenosteum (Coe) and corallites (Cor) are indicated in the figure by white arrows. The red dotted squares (1) and (2) indicate specific analysed regions (see below). Lighter color indicates higher concentration.

As mentioned previously, the coral sample that has been analysed comes from a *Dendrophyllia ramea* collected from the Bay of Cadiz (on the Atlantic coast in southwestern Spain). This analysis covered a region of approximately 7.5 cm × 2.5 cm (that is, a surface area of ~19 cm²) that was obtained from a longitudinal section of the coral skeleton. An optical image of the sample, as well as the corresponding distributions of Mg, Sr and Na, are shown in Fig. 5. Another possible representation is shown in Supplementary Figure 1.The complex growth mode and the inner structure of the dendroid colony is finely resolved by the elemental images. The structures of the coenosteum (Fig. 5, Coe) and the septa (Fig. 5) of each corallite (Fig. 5, Cor) are perfectly recognizable; the septa of the central branch can be traced easily. Variations in the Mg/Ca and Sr/Ca ratios are observed along the coral growth axis and were likely caused by the differential incorporation of trace elements due to climatic and environmental features, i.e., the high amount of Mg at the external edge of the coenosteum and the margins of the calices. Some isolated heterogeneities (i.e., in the septa) involving high amounts of Mg and Sr probably represent “vital effects” (i.e., centres of calcification, COCs), which are usually characterized by high concentrations of Mg, Sr, S, and Ba^28, 39^.

**Figure 6:**
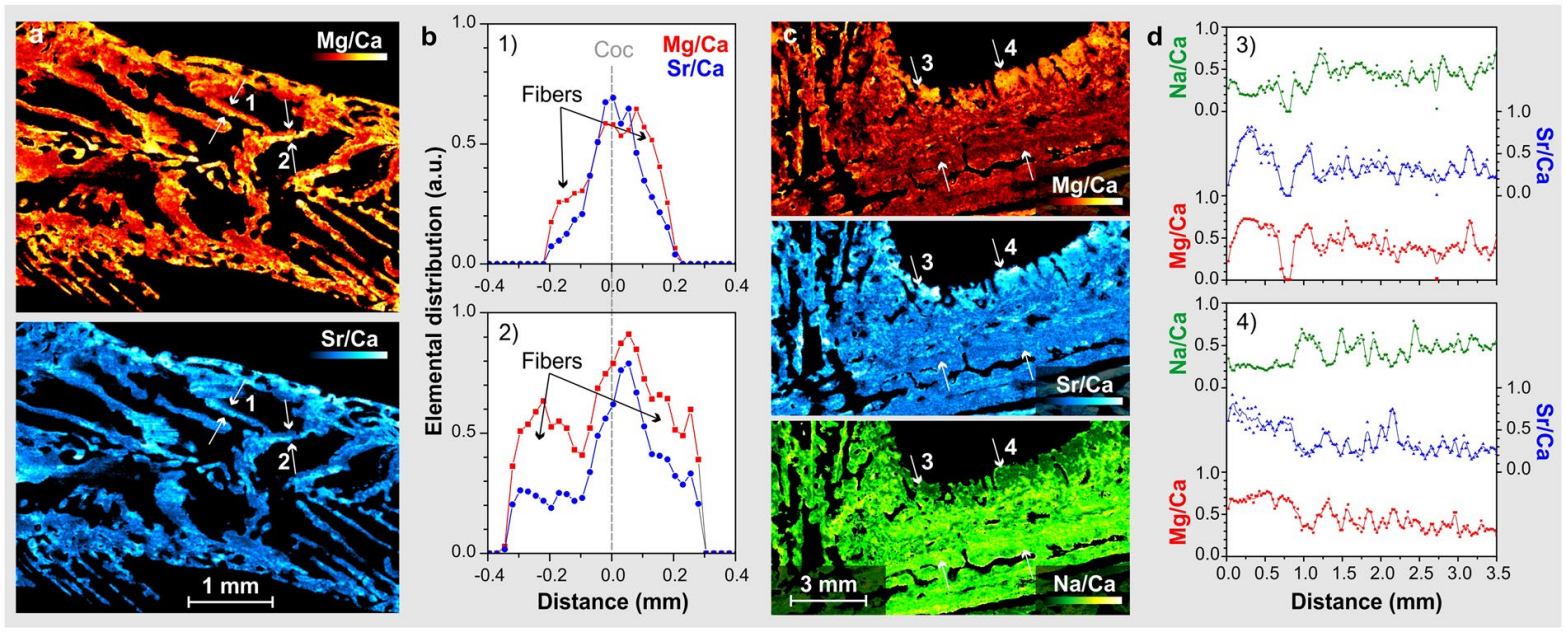
(**a**) Elemental images (Mg/Ca, Sr/Ca) of *Dendrophyllia* in the region represented by rectangle (1) in Fig. 5, which shows COC transects where fibres are located (**b**); (**c**) elemental images (Na/Sr/Mg) of the region represented by rectangle (2) in Fig. 5, with transverse profiles (**d**) across the coenosteum. Both images (**a**) and (**b**) has been tilted by 90° for better observation.

*Dendrophyllia* is a cold-water coral with a complex dendroid structure, and the choice of sampling strategy is critical in obtaining representative climatic records. It is common to avoid the COCs during sampling based on optical observation^24, 40^. In the case of *Dendrophyllia*, the septa have long COCs that cut the septa longitudinally. An example is shown in Fig. 6a,b where two different profiles have been extracted from a septum cutting a COC surrounded by fibres. The profiles of Mg/Ca and Sr/Ca show an uncorrelated distribution and are sharper for Sr/Ca than for Mg/Ca. The contrast between Mg and Sr amounts is very strong in the COCs versus the fibrous areas, a feature previously observed in other corals^28, 39^.

Calcification takes place in two main areas that have different growth rates, the coenosteum and the calices. The main branch shows continuous vertical growth that is faster than that of the lateral calices, whereas the coenosteum, the spongy tissue between corallites, exhibits slow lateral accretion. The walls of the central branch show compositional variations along the growth axis (Sr/Ca, Mg/Ca, Na/Ca) that probably record several decades of climatic and environmental information, in accordance with the slow calcification rate of *Dendrophyllia* ^30^. Two transverse profiles across the coenosteum were created (Fig. 6c,d), to assess the reproducibility of the technique. These profiles show a reproducible signature along the transverse profiles, which represents condensed series of paleoclimate information that are easily correlated. Correlation analysis was accomplished using the Pearson coefficient between the different ratios and profiles. Metal/Ca ratios show cyclical variations with high correlation coefficients in the first profile (Sr/Ca-Mg/Ca: 0.77; Sr/Ca-Na/Ca −0.81; Mg/Ca-Na/Ca: −0.77). The Mg/Ca correlation coefficient is lower, likely due to biological incorporation of Mg. In contrast, the second profile shows a very good correlation between Sr/Ca - Mg/Ca (0.80) and the Na/Ca ratio is more variable (Sr/Ca: −0.64; Mg/Ca: −0.65). The correlation coefficients and the similarities between two profiles indicate high reproducibility of the technique in this kind of sample.

## Discussion

Several micro-analytical imaging methods have previously been used to analyse the spatial distribution of minor and trace elements in speleothems and corals. These methods include inductively coupled plasma mass spectrometry (LA-ICP-MS)^33, 41–43^; electron microprobe analysis (EPMA)^34^; proton-induced X-ray emission (PIXE)^40^; secondary ionization mass spectrometry (SIMS)^18, 34, 37, 39, 44^; and synchrotron X-ray microprobe techniques^21, 33, 34, 39, 41–46^. Some of these techniques have high performance in terms of both resolution and detection limits (SIMS, µ-XRF). Others have high spatial resolution but lower sensitivity (EPMA), while still others have better limits of detection but lower resolutions (LA-ICP-based techniques). However, all of them are typically limited to small sample sizes (~cm²) and also generally require time-consuming sample preparation. These technologies are also generally limited in terms of their acquisition rate (~1–10 Hz/pixel). Probably the closer technique to LIBS imaging could be the SR-micro XRF Synchrotron-micro XRF, whose potential for analysing speleothems has been shown^21, 45^, being particularly powerful in the high resolution detection of some elements, such as strontium, and capable of generating maps of some square centimetres. SR-micro XRF mapping has also the advantage of providing elastic scattering maps, which are sensitive to defects in the structure of the mineral and thus allowing to investigate relationship between element distribution in the sample and its textural characteristics. SR-micro XRF and LIBS imaging techniques should not however considered as equivalent but complementary. The LIBS method herein can be developed in a standard laboratory at reasonable costs, in contrast to SR-micro XRF, which requires of access to the big scientific synchrotron installations. LIBS imaging yields a reasonable response in high resolution of a wide range of elements on much larger samples (>50 cm²) than at SR-micro XRF. and works notably faster.

This technology has no intrinsic limitations in terms of scanning speed, which is only governed by the laser repeat rate and the acquisition rates of the detector. Acquisition rates of the order of 1 kHz have already been demonstrated^11, 47, 48^. These advantages endow LIBS imaging with unique capabilities in the scanning of large sample surfaces. In addition, the full compatibility of LIBS instrumentation with optical microscopy and its ease of use make clear that this technique has high growth potential and carries the possibility to provide additional information in the near future. It is also simple to apply such imaging techniques in research laboratories.

As mentioned above, LIBS imaging technology appears highly attractive in the field of Earth science and in particular for paleoclimate studies, since it allows the variations in climate proxies to be characterized over a large scale covering several centuries and may provide climate information that reveals seasonal changes. However, the large amount of information that LIBS imaging can collect needs to fit with the standard requirements of paleoclimate studies, in particular the need to provide time series (i.e., variations in the different proxies through time). Microanalytical imaging techniques, which are applied to speleothems and corals, have been used to study local variations in paleoproxies (trace element and isotopic compositions) at scales below 1 mm^2^ to several micrometres in corals and several mm^2^ in speleothems^21, 37^. In the case of speleothems, LIBS imaging of large areas at high spatial resolution offers, for the first time, compositional and fabric information that could be translated to paleoclimate features. Additionally, these experiments allow understanding and easy discrimination of the climatic and environmental signatures of “vital effects” throughout the coral skeleton^28, 39^. Furthermore, the different growth modes and sizes of cold-water and tropical coral colonies makes it necessary to develop sampling strategies that allow extraction of the maximum paleoclimate information^24^. Megapixel LIBS imaging arises as a powerful analytical technique in the study of variations in trace elements at micro- and macroscales in coral and speleothem samples and in the definition of the limits of feasibility of trace elements as paleoclimate proxies. The retrieved data will allow delving into the processes controlling precipitation and bio-crystallization, as well as the factors controlling the spatio-temporal variability of trace elements in large samples from speleothems and corals. The high-resolution characterization of such variability and its understanding are key tasks in studies of paleoclimate and paleoenvironmental reconstructions. LIBS imaging represents a great step forward, and this work lays the foundations for future paleoclimate research in these and other common natural archives. The technique opens vast opportunities for generating new high-resolution paleoclimate records from relatively large samples of a wide range of ages and provenances. Future work should aim to improve and standardize the technique so that it can be incorporated into paleoclimate laboratories and research projects. Integrative approaches of LIBS-imaging and other techniques, particularly petrographic, textural and microstratigraphic analyses, should contribute to: 1) obtaining better multi-proxy records of paleoclimate resulting from the integration of different chemical data; 2) improved understanding of coral and speleothem growth patterns; and 3) identifying diagenetic overprints or altered and biologically influenced areas in samples (thus improving sampling strategies for other studies, such as isotopic geochemistry).

## Methods

### Sample preparation

#### Speleothem sample

Sample SLX 2B is a longitudinal polished slab from a stalagmite retrieved from Sílex Gallery in Cueva Mayor in Burgos Province, Spain (42°21.53′N, 3°31.1′W) with a permit given by Junta de Castilla y León (Spain) and the support of the Grupo Espeleológico Edelweiss (Exma. Dip. Prov. Burgos). The sample was considered for paleoclimate studies in the framework of the CLISP/RECCE research projects. It is stored in the Stratigraphy Department of the Complutense University of Madrid.

#### Coral sample

A coral branch of the species *Dendrophyllia ramea* belonging to the biggest colony in the Invertebrate Collection of the Museo Nacional de Ciencias Naturales (MNCN, Spain). The colony was collected alive from the Bay of Cadiz (Cadiz, Spain), Atlantic Ocean (36°36.76′-36°36.76′ N - 6°26.66′-6°26.65′ O) at a depth of 20–24 m. The sample belongs to the Fauna Ibérica Project-I (DGICYT. Ministerio de Educación y Ciencia) and was collected in 1989. The coral branch was cleaned with a water jet to remove possible organic tissues or colonizers. The sample was cleaned by immersion in a NaClO 4% solution to remove organic contaminants, such as endolithic organisms like fungi and algae, rinsed several times in an ultrasonic bath (3 minutes) with MilliQ H_2_O, and finally oven-dried at 35 °C overnight. The sample was embedded in epoxy resin before a thin section was prepared by cutting longitudinally along the growth axis.

#### Experimental LIBS setup and data acquisition

The instrumental setup was based on an optical microscope built by the authors that combined a LIBS laser injection line, a standard optical-imaging apparatus, and a three-dimensional motorized platform for sample positioning. LIBS experiments used fundamental Nd: YAG laser pulses of 1064 nm (Centurion, Quantel), vertically focused onto the sample by a 15x magnification objective (LMM-15X-P01, Thorlabs). The pulse duration was 5 ns and the repetition rate was 100 Hz. During the experiments, the sample could be translated along three axes by a x-y-z-motorized stage. For the y and z stages, the travel range was 50 mm, and the maximum speed was 3 mm/s. The travel range and the maximum speed were, respectively, 100 mm and 10 mm/s for the x stage. The measurements were performed at room temperature. The proposed autofocus system was activated during all experiments. The light emitted by the plasma plume was collected by two independent collection systems composed of a quartz lens (f = 2 cm) positioned at an angle of approximately 50° relative to the sample surface, and focused onto the entrances of optical fibre bundles composed of 19 fibres with a 200 µm core diameter each with a round to linear bundle in both cases. The linear ends of the fibres were connected to two Czerny-Turner spectrometers, a Shamrock 500 and a Shamrock 163 (both from Andor Technology), that were equipped with 600 l/mm and 1200 l/mm gratings, respectively. Both spectrometers were equipped with intensified charge-coupled device (ICCD) cameras. The ICCD cameras were synchronized with the Q-switch of the laser. In both cases, a delay of 800 ns and a gate of 3 µs were used. The width of the entrance slit of both spectrometers was set to 30 µm. The selected emission lines and associated wavelengths are summarized in Supplementary Table 1. Different spectrometer ranges could be chosen depending on the elements of interest. In this particular case, for the detection of C, Si, Fe, Mg, Na and Ca, a Shamrock 500 spectrometer was used, setting the central wavelength to approximately 290 nm. Second, an additional spectrometer (a Shamrock 163) was used for the detection of Mn, Sr and Ca, fixing the central wavelength to 407 nm. Using this configuration, a spectral measurement range between 70 nm and 30 nm was accessible, and both spectrometers had a spectral resolution of approximately 0.15 nm. To perform the mapping experiments at the greatest possible speed, the movement of the sample was synchronized with the laser firing. The sample surface was scanned line by line (i.e., in the x direction) to cover the region of interest. In this configuration, the step size could be adjusted by setting the speed of the x translation stage. The laser energy was stabilized throughout the experiment using a servo control loop to improve the long-term stability of the laser output. This loop was achieved by using a power metre and a computer-controlled attenuator (ATT1064, Quantum Composers). A typical energy of 900 µJ per pulse was used in all the experiments. Homemade software developed in the LabVIEW environment controlled the entire system.

#### Construction of elemental images

An advanced spectrum treatment was developed to perform a rapid intensity extraction for each measurement site and for each species of interest. A single emission line was selected for each element of interest, and the algorithm defined a baseline fit using a polynomial function and subtracted it from the emission signal. Emission lines were selected based on two criteria: each selected line was required to be the strongest line of each element in the probed range and to be unaffected by any possible interference from other lines. With this software, a typical image of ~3 million of spectra was processed in less than 5 minutes (time estimated for 1 dataset). A two-dimensional matrix containing the selected line intensity from a point on the sample surface was provided for each element and could be displayed using a false-colour scale to present a visual result in the form of elemental images. All images were then processed using ImageJ software (NIH, Bethesda, MD, www.nih.gov); the images were contrasted (if necessary, and only in a linear manner), slightly smoothed (using a 0.5-pixel Gaussian smoothing), and converted from 16-bit to 8-bit images. Note that all the Mg, Sr and Na images presented in this manuscript have been normalized to Ca. If necessary, a mask image retrieved from Ca was used to apply the normalization. The selected emission lines and associated wavelengths are summarized in Supplementary Table 1.

## Electronic supplementary material

Suplemantary File
