# Supplementary material for: Megapixel multi-elemental imaging by Laser-Induced Breakdown Spectroscopy, a technology with considerable potential for paleoclimate studies
Source: Sci Rep. 2017 Jul 11;7:5080. doi: 10.1038/s41598-017-05437-3 (PMC5505998; doi:10.1038/s41598-017-05437-3)
Supplement: Supplementary file 1 — Suplemantary File [file 41598_2017_5437_MOESM1_ESM.pdf]

Manuscript “Megapixel multi-elemental imaging by Laser-Induced Breakdown Spectroscopy, a technology with considerable potential for paleoclimate studies” by J. Caceres *et al.*

Full Author List

1.- J. O. Cáceres

2.- F. Pelascini

3.- V. Motto-Ros

4.- S. Moncayo

5.- F. Trichard

6.- G. Panczer

7.- A. Marín-Roldán

8.- J.A. Cruz

9.- I. Coronado

10.- J. Martín-Chivelet

Supplementary Table 1

Element	Specie	Wavelength (nm)
**Ca**	I	300.09
**Mg**	I	85.21
**Na**	I	330.30
**Sr**	II	407.77
**Si**	I	288.16
**Fe**	I	302.11
**Al**	I	309.27
**Mn**	I	403.31

Table S1: Lines used for the element of interest.

Supplementary Figure 1

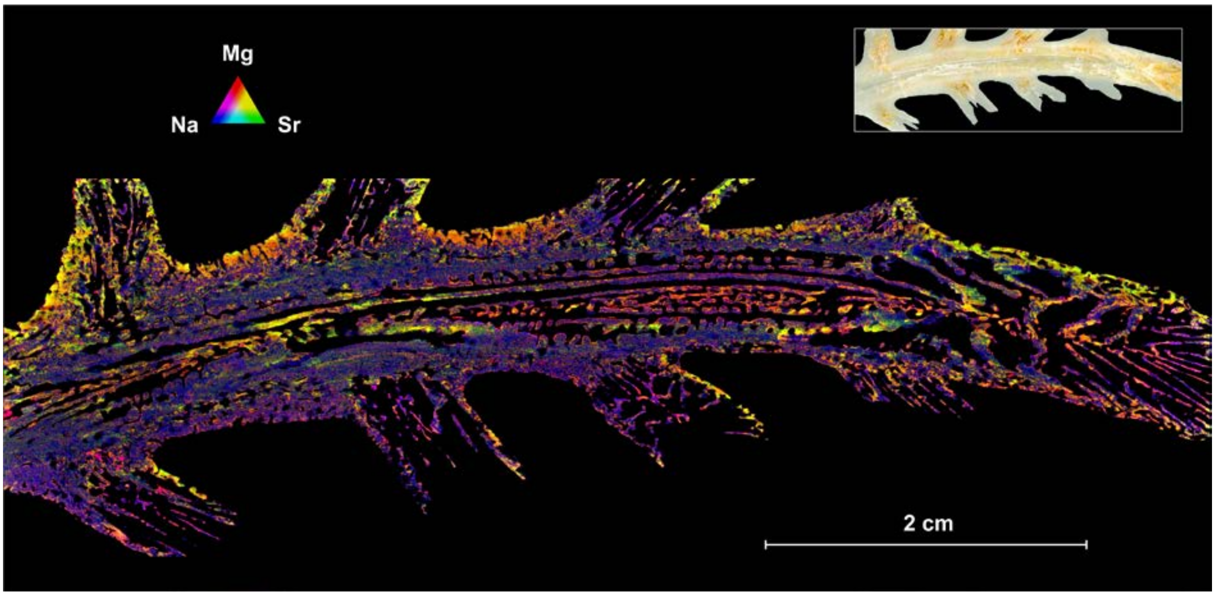

Figure S1: RGB (Red = Mg, Green = Sr, Blue = Sr) representation of the coral sample.
